# Serum Fatty Acid Profiles Are Associated with Disease Activity in Early Rheumatoid Arthritis: Results from the ESPOIR Cohort

**DOI:** 10.3390/nu14142947

**Published:** 2022-07-19

**Authors:** Johanna Sigaux, Alice Bellicha, Camille Buscail, Chantal Julia, René-Marc Flipo, Alain Cantagrel, Francois Laporte, Caroline Beal, Marie-Christophe Boissier, Luca Semerano

**Affiliations:** 1 Inserm U1125, Université Sorbonne Paris Nord, Li2P, 93000 Bobigny, France; johanna.siagaux@aphp.fr (J.S.); marie-christophe.boissier@aphp.fr (M.-C.B.); 2 Hôpital Avicenne Service de Rhumatologie, GHU-PSSD, Assistance Publique-Hôpitaux de Paris, 93000 Bobigny, France; caroline.beal@aphp.fr; 3 Équipe de Recherche en Épidémiologie Nutritionnelle (EREN)—Inserm U1153, Inrae U1125, Cnam, Centre de Recherche Epidémiologie et Statistiques (CRESS), Université Sorbonne Paris Nord, 93000 Bobigny, France; a.bellicha@eren.smbh.univ-paris13.fr (A.B.); c.buscail@eren.smbh.univ-paris13.fr (C.B.); julia@eren.smbh.univ-paris13.fr (C.J.); 4 Public Health Department, GHU-PSSD, Assistance Publique-Hôpitaux de Paris (APHP), 75004 Bobigny, France; 5 Service de Rhumatologie, Université de Lille, CHU de Lille, 59037 Lille, France; rene-marc.flipo@chru-lille.fr; 6 Centre de Rhumatologie, CHU de Toulouse, 31059 Toulouse, France; cantagrel.a@chu-toulouse.fr; 7 Département de Biochimie-Pharmacologie-Toxicologie, CHU de Grenoble, 38700 Grenoble, France; flaporte@chu-grenoble.fr

**Keywords:** omega 3 fatty acids, *n*-3 fatty acids, omega 6 fatty acids, *n*-6 fatty acids rheumatoid arthritis, cohort study

## Abstract

Background: Long-chain omega-3 and omega-6 fatty acids (*n*-3, *n*-6 FAs) may modulate inflammation and affect the risk of developing rheumatoid arthritis (RA). However, whether *n*-3/*n*-6 FA status affects RA after disease onset is unknown. This study aimed to assess whether FA profiles are independently associated with disease activity in a large prospective cohort of patients with early RA. Methods: Baseline serum FAs were quantified in 669 patients in the ESPOIR cohort. Principal component analysis identified three serum FA patterns that were rich in *n-*7–9, *n*-3 and *n*-6 FAs (patterns ω7–9, ω3 and ω6), respectively. The association of pattern tertiles with baseline variables and 6-month disease activity was tested using multivariable logistic regression. Results: Pattern ω3 was associated with low baseline and pattern ω6 with high baseline *C*-reactive protein level and disease activity. Both patterns ω3 and ω6 were associated with reduced odds of active disease after 6 months of follow-up (pattern ω3: odds ratio, tertile three vs. one, 0.49 [95% CI 0.25 to 0.97] and pattern ω6: 0.51 [0.28 to 0.95]; *p* = 0.04 and 0.03, respectively). Conclusions: In a cohort of early RA patients, a serum lipid profile rich in *n*-3 FAs was independently associated with persistently reduced disease activity between baseline and 6-month follow-up. An *n*-6 FA profile was also associated with lower 6-month disease activity.

## 1. Introduction

Rheumatoid arthritis (RA) is a multifactorial chronic inflammatory disease characterized by joint inflammation and the production of autoantibodies like rheumatoid factor (RF) and anti-citrullinated peptide antibodies (ACPAs) [1]. Genetic susceptibility is estimated to account for 40% to 50% of the risk of developing RA [2]. The residual risk is generally attributed to environmental factors that may act on predisposed individuals before disease onset. This hypothesis supposes the existence of a preclinical phase of RA [3] characterized by the presence of disease-specific autoantibodies, with no evidence of joint inflammation [4]. Thus, susceptible individuals may or may not progress to RA depending on exposure to environmental factors not clearly identified yet, apart from smoking and silica exposure [5]. After disease onset, these factors may concur to determine the disease phenotype and prognosis. Thus, the identification of additional environmental factors involved in the pathogenesis of RA may help prevent RA and reduce its severity.

Long-chain *n*-3 and *n*-6 polyunsaturated fatty acids (PUFAs; *n*-3, *n*-6 FAs) are involved in immune homeostasis and can alter inflammatory processes [6]. Long-chain PUFAs cannot be efficiently synthesized by humans and need to be introduced with diet [7]. Fatty fish is the main dietary source of long-chain *n*-3 PUFAs, eicosapentaenoic acid (EPA) (20:5*n*-3) and docosahexaenoic acid (DHA). EPA and DHA may also be produced by elongation of the plant-derived alpha linolenic acid (ALA) (18:3*n*-3). However, the conversion to EPA, and even more to DHA, is inefficient, especially in humans, and is antagonized in the presence of the more abundant *n*-6 FAs in the diet that compete for the involved enzymes delta-5 and -6 desaturases [8,9]. Seeds and vegetable oils (e.g., sunflower or corn) are widespread sources of the *n*-6 PUFA linoleic acid (LA) (18:2*n*-6). Unlike the *n*-3 homologous ALA, LA is efficiently converted to arachidonic acid (20:4*n*-6) and gamma-LA (GLA) (18:4*n*-6).

Although *n*-6 FAs are considered mainly proinflammatory, some, like GLA, may be endowed with anti-inflammatory activity [10], and in a nested case–control study, the erythrocyte content in LA was found inversely associated with risk of RA [11].

Numerous mechanistic studies support that *n*-3 FAs, particularly EPA and DHA, may reduce inflammation and the immune response via different potential mechanisms, such as the production of anti-inflammatory lipid mediators [12,13], reduced production of pro-inflammatory cytokines [14,15] or induction of a regulatory phenotype in immune cells [16]. A modest, but quite consistent, clinical effect of fish oil/*n*-3 FAs supplementation has been described in RA [17,18].

Dietary intake of *n*-3 FAs (by supplementation or fish consumption) has been found inversely associated with the incidence of RA in different populations [19,20,21]. However, that association has not been consistently found [22,23], likely because fish consumption is correlated only partially with in vivo levels of *n*-3 FAs. The latter levels are also influenced by interindividual heterogeneity in the absorption and metabolism of FAs [24]. Thus, in a recent nationwide randomized controlled trial, 1 g per day of *n*-3 FA supplementation for a mean of 5.3 years resulted in a 15% non-statistically significant reduction in incidence of autoimmune diseases versus placebo [25].

Directly measuring PUFA levels (in plasma, serum or red blood cell (RBC) membrane) may provide a more valid biomarker of PUFA status for use in epidemiological studies. Accordingly, three nested case–control studies in the same cohort found that in individuals genetically at risk of RA, high RBC membrane levels of *n*-3 FAs were inversely associated with ACPA positivity [26], with a stronger association in shared-epitope carriers [27], and that high levels of docosapentaenoic acid and DHA were associated with lower transition from asymptomatic autoimmunity to undifferentiated arthritis [28]. In a case–control study of women with early RA, levels of RBC, EPA and DHA were associated with reduced odds of RA [29].

Conversely, it is still unclear, particularly in the early phases of RA, whether FA status may affect patient features and disease evolution. Moreover, few studies [29], and none with a longitudinal design, have analyzed a wide panel of FAs, which may better reflect the interplay between FAs with different biological activity.

In this study, we hypothesized that patients with RA may have different disease features and prognosis depending on their FA status. Thus, we characterized serum patterns of FAs in a large cohort of patients with early RA and estimated the association of those patterns with patient features and with baseline and 6-month disease activity.

## 2. Materials and Methods

### 2.1. Study Population

The characteristics and criteria of enrollment of patients in the ESPOIR cohort (Etude et Suivi des POlyarthrites Indifférenciées Récentes) are detailed elsewhere [30]. Briefly, ESPOIR is a national French, multicentric (14 rheumatology centers), longitudinal cohort of patients 18 to 70 years old with suspected or confirmed diagnosis of RA. Patients who had two or more swollen joints for 6 weeks or 6 months were included in the cohort between 2002 and 2005, with a planned follow-up of 15 years. The follow-up visits with collection of clinical biological and radiological data were scheduled every 6 months during the first year and yearly thereafter. All 813 patients included in the cohort provided informed consent, and the study was approved by the Montpellier Ethics Committee in July 2002 (no. 020307). For this study, we analyzed data for 669 patients with early arthritis included in the ESPOIR cohort who fulfilled the 2010 ACR/EULAR criteria at enrollment in the cohort or during the first 2 years of follow-up.

### 2.2. FA Analysis

At enrollment in the cohort (from December 2002 to March 2005), all participants underwent fasting blood draw. Serum samples were stored at −80 °C in a single center facility (Biological Resources Center at Bichat Hospital, Paris). Methyl esters of different FAs were quantitatively analyzed to measure serum FA composition among serum lipids, as described elsewhere [31]. Lipids were extracted from 100-μL aliquots of serum with 8 μL hexane:isopropanol (3:2, vol:vol) in the presence of heptadecanoic acid as an internal standard. The protocol reports that methylated FAs, obtained by standard saponification of triglycerides and cholesterol esters followed by methylation with boron trifluoride 14% in methanol and extraction with *n*-heptane, are dosed by gas chromatography with a flame ionization detector (6850 Agilent) by using a capillary column (Quadrex 30 m length, 0.25 mm id, film thickness 0.25 mm, Alltech), with H_2_ as a carrier gas. A typical run uses a temperature gradient of 50 uC/min from 50 uC to 140 uC, 1.4 uC/min from 140 uC to 165 uC and 10 uC/min from 165 uC to 245 uC. Individual methyl esters were identified by comparison with a mixture of commercial standards, with quantification expressed both as serum concentration (μmol/L) and as percentage total area of all FA peaks.

### 2.3. Statistical Analyses

We extracted FA patterns from 19 serum FAs by using principal component analysis (PCA) [32]. PCA allowed for generating FA patterns (principal components or factors) as independent linear combinations of the original 19 serum FAs, using orthogonal transformation (Varimax option) to obtain independent patterns. We determined the number of patterns to retain for the analysis on the basis of each factor’s eigenvalue and according to Cattel’s scree test (a plot of the total variance explained by each pattern) [33]. We named each pattern according to the FA content correlation with each pattern by using Pearson correlation coefficients. Patients were classified by tertiles of each FA pattern score.

Univariate associations between tertiles of FA patterns and categorical patient variables were tested with chi-squared test or trend tests depending on the type of variable. The association with continuous variables was tested with ANOVA or Kruskal–Wallis test according to the data distribution.

For multivariable analysis, a logistic regression model was used to estimate odds ratios (ORs) with *p* for trend calculation across tertiles of FA patterns (as ordinal explicatory variables) and Disease Activity Score in 28 joints (DAS28) based on erythrocyte sedimentation rate (ESR) by using the 5.1 threshold for high disease activity as the categorical dependent variable. Additional analyses were performed with lower thresholds of the DAS28 (3.2 and 2.6, lower values indicating low disease activity and remission, respectively). Hence, the final models included age, sex, body mass index, smoking status, education level, profession, baseline ACPA and RF status, baseline treatments (nonsteroidal anti-inflammatory drugs, corticosteroids, statins, hormone replacement therapy or oral contraception, and beta blockers), and baseline *C*-reactive protein (CRP) level. Baseline DAS28 and conventional or biologic disease-modifying anti-rheumatic drug (c or bDMARD) treatment between baseline and 6 months were also included in the models to study the association between tertiles of FA patterns and 6-month disease activity. Sensitivity analyses involved excluding baseline CRP level from the main models. All statistical analyses were performed with SAS^®^ software version 9.4 (SAS Institute Inc., Cary, NC, USA). 

## 3. Results

### 3.1. Study Population

The main characteristics of the study population are in Table 1. The patients were mostly females (77.3%) and of Caucasian origin; about half were never smokers. At the time of inclusion in the cohort, 47.1% of patients were ACPA-positive and 57.6% were RF-positive. The mean (SD) DAS28 at inclusion was 5.2 (1.3), thus indicating overall high disease activity, and 15.0% of patients already had erosive disease. Only 18.1% had received oral corticosteroids; most had not received cDMARDs (92.2%) and none had received bDMARDs. At 6-month follow-up, 75% of patients had received cDMARDs and 3% bDMARDs. The mean DAS28 at 6 months was 3.3 (1.4).

### 3.2. Serum FA Quantification and Pattern Definition

Serum FA analysis for the 669 patients allowed for quantifying a total of 19 FAs with chain length from 14 to 22 carbon atoms (Table 2). Six were *n*-6 PUFAs, five were *n*-3 PUFAs, two were *n*-7 PUFAs, two were *n*-9 PUFAs and four were saturated FAs. We used PCA to extract patterns of FAs that could capture the maximum variance in FA content between patients. The first pattern was called pattern ω7–9 because it featured higher positive correlations with *n*-7 (palmitoleic and vaccenic) and *n*-9 (oleic) PUFAs, together with saturated FAs (myristic and palmitic). The second pattern was called pattern ω3 because it featured high positive correlations with *n*-3 long-chain PUFAs (EPA, docosapentaenoic acid and DHA) and negative correlations with *n*-6 PUFAs (C20_2n6 and C20_3n6). The third pattern was called pattern ω6 and was rich in *n*-6 long-chain FAs (GLA, di-homo-gamma-LA, eicosatrienoic acid). The pattern correlations with individual FAs are in Table 2.

### 3.3. Associations between FA Patterns and Patient Features at Baseline

At baseline, high pattern ω3 scores were associated with low CRP level, less corticosteroid treatment and less high disease activity (DAS28 > 5.1) (Table 3). High pattern ω6 scores were associated with smoking, increased BMI, low educational level, ACPA positivity, erosive disease and high CRP level and disease activity (Supplementary Table S1). High pattern ω7–9 scores were associated with smoking, high BMI and Caucasian ethnicity. Both patterns ω7–9 and ω6 were associated with increased concomitant use of statins and beta-blockers (Supplementary Tables S1 and S2). Pattern ω7–9 was associated with increased use of hormone replacement therapy or oral contraception (Supplementary Table S2) and both patterns ω3 and ω6 were associated with reduced use (Table 3 and Table S1).

We then tested the associations between FA patterns and baseline disease activity on multivariable analysis. In the model not adjusted on CRP level, high pattern ω3 scores were associated with reduced odds of high baseline disease activity (DAS28 ≥ 5.1) (Table 4). The association did not remain when CRP level was included (Table 4), which suggests that CRP may be a path variable in the association between FAs and concomitant disease activity. We found no significant associations between ω7–9 and ω6 patterns and high disease activity on multivariable analysis. 

### 3.4. Associations between FA Patterns and Patient Features at 6-Month Follow-Up

On univariate analysis, high pattern ω3 scores at baseline were associated with reduced odds of high 6-month disease activity (Supplementary Table S3). A similar association was found for pattern ω6 although not significant (Supplementary Table S3).

On multivariate analysis, higher tertiles of patterns ω3 and ω6 were associated with reduced odds of high 6-month disease activity (DAS28 ≥ 5.1) (Table 5). Those associations were unchanged when excluding CRP level from the model (Table 5). FA patterns were not associated with moderate to high disease activity (DAS28 ≥ 3.2) (Supplementary Table S4). However, we found a trend for higher odds of remission for high pattern ω3 scores (Supplementary Table S5).

## 4. Discussion

In a large cohort of patients with early RA, FA patterns, obtained from baseline serum samples, were associated with concomitant disease activity and severity and with further disease activity at 6-month follow-up, with differing directions depending on the FA pattern.

To the best of our knowledge, this is the largest study that measured FAs in patients with early RA in relation to disease features and the first to measure those associations prospectively. Moreover, this is the first cohort study to measure all FAs in serum (saturated and unsaturated, *n*-3, *n*-6, *n*-7 and *n*-9), providing, for a large number of patients, a thorough panel of FAs that might be involved in the inflammatory process in RA. 

To capture and summarize the complexity of potential interplay between different FAs, we extracted patterns of FAs. We adopted a data-driven approach, with no prior hypothesis, which led to identifying three independent patterns correlated more specifically with *n*-3 (pattern ω3), *n*-6 (pattern ω6), *n*-7–9 and saturated (pattern ω7–9) FAs, respectively.

Pattern ω3 was associated with reduced inflammation and disease activity both at baseline and at 6-month follow-up, whereas pattern ω6 was associated with increased inflammation and disease activity at baseline but also with reduced odds of high disease activity at 6 months. Pattern ω7–9 did not show any association with disease activity at those times.

In deeper detail, at baseline, pattern ω3 was associated with reduced CRP level and low to moderate disease activity. With the baseline inverse association between pattern ω3 and CRP level, we wondered whether CRP level might be an intermediate variable in the causal process by which PUFAs affect disease activity. Reverse causality (i.e., the inflammatory state affects the FA pattern and not the reverse) in these cross-sectional analyses, may be an alternative explanation. However, high pattern ω3 scores were also associated with low to moderate disease activity at 6-month follow-up in models including and excluding baseline CRP level.

Evidence from the literature suggests a potential role for *n*-3 FAs in reducing the risk of developing RA by interacting with the genetic background [26,27,28]. Case–control studies support that patients with RA may have lower content of long-chain *n*-3 FAs as compared with controls [29,34]. However, no longitudinal cohort study has evaluated the association between measured *n*-3 FA content and disease features and evolution in patients in the early phases of RA.

This study suggests that a profile high in *n*-3 FAs may characterize patients who are less likely to have high levels of disease activity over time.

In this cohort, a pattern high in *n*-6 FAs was associated with highly active disease and poor prognostic factors at baseline but also, interestingly, with low to moderate 6-month disease activity. This apparently discordant result needs to be further examined.

The models we used were adjusted on treatments, so more intensive treatment for patients with high pattern ω6 scores is not the most likely explanation. Index event bias [35,36] may potentially explain this result, however the association was independent of CRP level and baseline disease activity and therefore not restricted to patients who already exhibited high disease activity at inclusion in the cohort.

A biological explanation for our findings may rely on the potential anti-inflammatory effects of some *n*-6 FAs. Increased content of the *n*-6 PUFA LA in the RBC membrane was found associated with reduced risk of developing RA in the EPIC cohort [11]. In a cross-sectional study of patients with confirmed RA, a dietary pattern characterized by high consumption of vegetable-derived FA (ALA and LA) was associated with reduced risk of active disease [37]. However, LA was not a main contributor to pattern ω6 in this study. Conversely, both GLA and dihomo-GLA (DGLA) had high factor loadings for the pattern in our patients. In vitro studies showed that DGLA can inhibit human synovial cell proliferation [38] and T-cell early-activation gene expression [39]. Both GLA [40] and DGLA [41] can reduce tumor necrosis factor-alpha production in peripheral blood mononuclear cells, and GLA also showed some clinical efficacy in RA [42,43]. Recently, a panel of peripheral-blood specialized pro-resolving mediators derived from *n*-3, but also *n*-6 FAs was found associated with response to methotrexate in a cohort of early RA [44]. 

Alternatively, baseline FA profile may have limited predictivity in the long term. FAs were quantified only at baseline, and we do not know whether those patterns were stable over time. An additional issue concerning the stability of the patterns is that serum may not be the most appropriate source for analysis of FAs. The FA content of the RBC membrane is considered the gold standard for FA analysis. Although RBC and plasma (or serum) FA concentrations are highly correlated, serum FA content seems better correlated with (and therefore influenced by) recent dietary intake than RBC FA content [45]. The lifespan of RBCs is longer than that of lipoproteins, so RBC FAs may reflect a more stable steady state, acquired over months, whereas plasma FAs may reflect the dietary intake over weeks. For example, *n*-3 FA EPA content reaches the steady state in 4 to 8 weeks in plasma and 180 days in RBCs [46]. Hence, in this study, FA patterns may better reflect the state of the patient at the time of the measurement. Moreover, it has been reported that people diagnosed with RA and other chronic inflammatory diseases often change their dietary habits after the diagnosis [47], which may have modified the FA profiles during the first months of follow-up in the cohort. Algorithms of conversion of FA percentages from plasma to RBCs have been published [48] and may be used to compare results of studies with different FA quantification methods.

The abovementioned issues are the main limitations of this study. The strengths are the longitudinal design, the size of the cohort, and the completeness of data that allowed for adjusting the statistical models on a number of potential confounders (although residual undetected confounding cannot be ruled out). An additional strength is measurement of the panel of all FAs in the largest number of RA patients in the literature. Nevertheless, although the patterns allowed for capturing the overall FA composition better than the analysis of single FAs, this study evaluated the potential influence on RA of each pattern separately. Hence, we cannot exclude that an “ideal” FA profile in RA patients may exist, which might result from a combination of FAs from the three patterns at different levels.

## 5. Conclusions

A thorough analysis of baseline serum contents of FAs in a large cohort of patients with early RA identified a lipid signature enriched in *n*-3 FAs that was independently associated with persistently low to moderate disease activity between inclusion in the cohort and 6-month follow-up. A pattern characterized by *n*-6 FAs was also associated with low to moderate 6-month disease activity.

## Figures and Tables

**Table 1:** Demographic and disease characteristics of patients with early rheumatoid arthritis (RA) from the ESPOIR cohort (*n* = 669).

	*n* (%)	Mean (SD) or Median (Range)
**Anthropometrics**		
Age (years)	-	48.6 (12.3)
Sex		
Male	152 (22.7)	-
Female	517 (77.3)	-
Ethnicity		
Caucasian	615 (91.9)	-
Other	54 (8.1)	-
BMI (kg/m^2^)		
<25.0	391 (58.6)	-
25.0–30.0	180 (27.0)	-
>30.0	96 (14.4)	-
**Sociodemodemographic Variables**		
Marital status		
Married *	486 (72.8)	-
Single ^†^	182 (27.3)	-
Education level		
None to primary school	306 (45.7)	-
Secondary school	152 (22.7)	-
Tertiary education or higher	211 (31.5)	-
Professional status		
Active or student	442 (66.1)	-
Unable to work	10 (1.5)	-
Housewife/husband	48 (7.1)	-
Unemployed	32 (4.8)	-
Retired	136 (20.4)	-
Smoking status		
Never smoker	355 (53.1)	-
Former smoker	172 (25.7)	-
Current smoker	142 (21.2)	-
**Laboratory Features**		
CRP	-	9.0 (0–384)
ESR		29.9 (25.0)
ACPA- and RF-positive	281 (47.3)	-
ACPA-positive/RF-negative	30 (5.1)	-
ACPA-negative/RF-positive	30 (5.1)	
ACPA-negative/RF-negative	253 (42.6)	
**Treatment For Ra**		
DMARDs at baseline		
None	617 (92.2)	-
cDMARDs	52 (7.8)	-
DMARDs at 6 months		
None	139 (22.0)	-
cDMARDs	475 (75.0)	-
bDMARDs	19 (3.0)	-
Oral corticosteroids at baseline		
None	548 (81.9)	-
Yes	121 (18.1)	-
**Ra Activity and Severity**		
DAS28 at baseline	-	5.2 (1.3)
DAS28 at 6 months	-	3.3 (1.4)
DAS28 > 5.1		
At baseline	-	340 (50.8)
At 6 months	-	84 (12.6)
Typical erosions at baseline		
Yes	-	100 (15.0)
No	-	568 (85.0)

* Married or in couple relationship. ^†^ Single, divorced or widowed. Data are mean ± SD or number (%). BMI: body mass index; CRP: *C*-reactive protein; DAS28: Disease Activity Score in 28 joints; ACPA: anti-citrullinated peptide antibodies; RF: rheumatoid factor (IgM); c/bDMARD: conventional/biological disease-modifying anti-rheumatic drugs.

**Table 2:** Patterns of fatty acids (FAs) and contribution of each FA to the pattern (correlation coefficients) (*n* = 669).

FA Omega Name	FA Common Name	Pattern ω7-9	Pattern ω3	Pattern ω6
		Pearson’s correlation coefficients		
C14_0	Myristic acid	0.52641		
C16_0	Palmitic acid	0.81581	−0.21467	
C16_1n7	Palmitoleic acid	0.79217		0.22254
C18_0	Stearic acid			0.36009
C18_1n7	Vaccenic acid	0.59262		
C18_1n9	Oleic acid	0.76549		
C18_2n6	Linoleic acid	−0.88203	−0.25486	−0.24690
C18_3n3	Alpha-linolenic acid		0.20374	−0.28524
C18_3n6	Gamma-linolenic acid			0.80262
C18_4n3	Stearidonic acid		0.38059	0.31487
C20_0	Arachidic acid			
C20_2n6	Eicosadienoic acid		−0.56736	
C20_3n6	DGLA		−0.28721	0.60980
C20_3n9	Mead’s acid (eicosatrienoic)	0.40752		0.68549
C20_4n6	Arachidonic acid	−0.35614	0.30190	0.48385
C20_5n3	EPA		0.86464	
C22_4n6	Docosatetraenoic acid			0.71141
C22_5n3	DPA		0.78835	
C22_6n3	DHA		0.77720	−0.22137

Pearson correlation method. DGLA: dihomo-gamma-linolenic acid; EPA: eicosapentaenoic acid; DPA: docosapentaenoic acid; DHA: docosahexaenoic acid.

**Table 3:** Univariate analysis of the association between pattern ω3 and patient characteristics at inclusion in the cohort.

Pattern ω3		T1	T2	T3	*p*
Age, mean (SD)	-	43.6 (12.8)	50.0 (11.8)	52.2 (10.5)	**<0.001**
Smoking	Never smoker	112 (50.2)	124 (55.6)	119 (53.4)	0.09
	Former smoker	52 (23.3)	64 (28.7)	56 (25.1)	
	Current smoker	59 (26.5)	35 (15.7)	48 (21.5)	
BMI	Underweight or normal	136 (61.0)	113 (51.1)	142 (63.7)	0.08
	Overweight	55 (24.7)	70 (31.7)	55 (24.7)	
	Obesity	32 (14.4)	38 (17.2)	26 (11.7)	
Ethnicity	Other	19 (8.5)	18 (8.1)	17 (7.6)	0.94
	Caucasian	204 (91.5)	205 (91.9)	206 (92.4)	
Sex	Men	50 (22.4)	56 (25.1)	46 (20.6)	0.52
	Women	173 (77.6)	167 (74.9)	177 (79.4)	
Professional status	Working or student	148 (66.4)	131 (59.0)	128 (57.4)	**0.04**
	Unemployed (whatever the cause)	45 (20.2)	40 (18.0)	40 (17.9)	
	Retired	30 (13.5)	51 (23.0)	55 (24.7)	
Education level	Primary school	96 (43.1)	116 (52.0)	94 (42.2)	0.05
	High school	62 (27.8)	41 (18.4)	49 (22.0)	
	Graduate education	65 (29.2)	66 (29.6)	80 (36.9)	
Marital status	Single	64 (28.7)	51 (23.0)	67 (30.0)	0.21
	Married	159 (71.3)	171 (77.0)	156 (70.0)	
ACPA	Positive	103 (46.8)	110 (50.2)	98 (44.1)	0.44
IgM-RF	Positive	120 (53.8)	130 (58.3)	135 (60.5)	0.34
Typical RA erosions	Yes	26 (11.7)	41 (18.5)	33 (14.8)	0.13
Corticosteroids	Yes	46 (20.6)	52 (23.3)	23 (10.3)	**<0.001**
NSAIDs	Yes	207 (92.8)	197 (88.3)	201 (90.1)	0.27
Beta-blockers	Yes	13 (5.9)	19 (8.5)	19 (8.5)	0.47
Statins	Yes	8 (3.6)	16 (7.2)	20 (9.0)	0.07
HRT or OC	Yes	57 (25.7)	32 (14.4)	23 (10.3)	**<0.001**
Baseline use of DMARDs	Yes	16 (7.2)	22 (9.9)	14 (6.3)	0.34
Baseline DAS28	>5.1 (high)	130 (59.1)	101 (46.5)	109 (49.8)	**0.02**
CRP, mean (SD)	-	25.5 (40.8)	19.2 (30.9)	17.6 (28.1)	**0.03**

Data are *n* (%) unless otherwise indicated. Bold *p* values are significant (*p* < 0.05). T1, T2, T3: tertile one, two and three of pattern score, respectively; BMI: body mass index; CRP: *C*-reactive protein; DAS28: Disease Activity Score in 28 joints based on ESR; HRT: hormone replacement therapy; OC: oral contraception; RA: rheumatoid arthritis; ACPA: anti-citrullinated peptide antibodies; RF: rheumatoid factor (IgM); c/bDMARD: conventional/biological disease-modifying anti-rheumatic drugs; NSAIDs: non-steroidal anti-inflammatory drugs.

**Table 4:** Multivariate analysis of the association between FA patterns and baseline high disease activity (DAS28 ≥ 5.1) (*n* = 669).

		T1	T2	T3	*p* Trend
**Pattern** **ω7–9**					
OR (95% CI)	Model 1	Ref	1.12 (0.74–1.72)	1.31 (0.85–2.03)	0.2
	Model 2	Ref	1.16 (0.78–1.72)	1.16 (0.78–1.72)	0.06
**Pattern** **ω3**					
OR (95% CI)	Model 1	Ref	0.56 (0.36–0.86)	0.69 (0.44–1.09)	0.1
	Model 2	Ref	0.49 (0.32–0.74)	0.61 (0.40–0.93)	0.02
**Pattern** **ω6**					
OR (95% CI)	Model 1	Ref	0.57 (0.37–0.88)	0.95 (0.61–1.47)	0.8
	Model 2	Ref	0.60 (0.40–0.90)	1.13 (0.74–1.68)	0.63

Model 1: adjusted for age, sex, BMI, smoking status, education level, work, baseline ACPA status, baseline RF, baseline treatments (NSAIDs, corticosteroids, statins, HRT or OC, beta blockers), baseline CRP level. Model 2: model 1 not adjusted for baseline CRP level. BMI: body mass index; CRP: *C*-reactive protein; HRT: hormone replacement therapy; OC: oral contraception; ACPA: anti-citrullinated peptide antibodies; RF: rheumatoid factor (IgM); NSAIDs: nonsteroidal anti-inflammatory drugs; OR: odds ratio; 95% CI: 95% confidence interval. T1, T2, T3: tertile one, two and three of pattern score, respectively.

**Table 5:** Multivariate analysis of the association between FA patterns and 6-month high disease activity (DAS28 ≥5.1) (*n* = 669).

		T1	T2	T3	*p* Trend
**Pattern** **ω7-9**					
OR (95% CI)	Model 1	Ref	0.99 (0.54–1.82)	0.80 (0.42–1.52)	0.5
	Model 2	Ref	0.99 (0.54–1.82)	0.79 (0.42–1.49)	0.5
**Pattern** **ω3**					
OR (95% CI)	Model 1	Ref	0.73 (0.40–1.34)	0.49 (0.25–0.97)	0.04
	Model 2	Ref	0.75 (0.41–1.38)	0.51 (0.26–1.00)	0.05
**Pattern** **ω6**					
OR (95% CI)	Model 1	Ref	0.43 (0.23–0.81)	0.51 (0.28–0.95)	0.03
	Model 2	Ref	0.43 (0.23–0.81)	0.51 (0.27–0.93)	0.02

Model 1: adjusted for age, sex, BMI, smoking status, education level, work, baseline ACPA status, baseline RF, baseline DAS28, baseline CRP level, baseline treatments (NSAIDs, corticosteroids, statins, HRT or OC, beta blockers, DMARDs treatment between 0 and 6 months. Model 2: model 1 not adjusted for baseline CRP level. BMI: body mass index; CRP: *C*-reactive protein; HRT: hormone replacement therapy; OC: oral contraception; ACPA: anti-citrullinated peptide antibodies; RF: rheumatoid factor (IgM); NSAIDs: nonsteroidal anti-inflammatory drugs; OR: odds ratio; 95% CI: 95% confidence interval.

## Data Availability

The data presented in this study are available on request from the corresponding author.

## Supplementary Materials

The following are available online at https://www.mdpi.com/article/10.3390/nu14142947/s1: Table S1. Univariate analysis of the association between pattern ω6 and patient characteristics at inclusion in the cohort; Table S2. Univariate analysis of the association between pattern ω7–9 and patient characteristics at inclusion in the cohort; Table S3. Univariate analysis of the association between baseline FA patterns and 6-month high disease activity (DAS28 >5.1) (*n* = 594); Table S4. Multivariate analysis of the association between FA patterns and 6-month moderate to high disease activity (DAS28 ≥ 3.2) (*n* = 594); Table S5. Multivariate analysis of the association between FA patterns and 6-month non-remission (DAS28 ≥ 2.6) (*n* = 594).

## References

[1] Boissier M.C., Biton J., Semerano L., Decker P., Bessis N. (2020). Origins of rheumatoid arthritis. Joint Bone Spine.

[2] Frisell T., Saevarsdottir S., Askling J. (2016). Family history of rheumatoid arthritis: An old concept with new developments. Nat. Rev. Rheumatol..

[3] Gerlag D.M., Raza K., van Baarsen L.G., Brouwer E., Buckley C.D., Burmester G.R., Gabay C., Catrina A.I., Cope A.P., Cornelis F. (2012). EULAR recommendations for terminology and research in individuals at risk of rheumatoid arthritis: Report from the Study Group for Risk Factors for Rheumatoid Arthritis. Ann. Rheum. Dis..

[4] Deane K.D., Demoruelle M.K., Kelmenson L.B., Kuhn K.A., Norris J.M., Holers V.M. (2017). Genetic and environmental risk factors for rheumatoid arthritis. Best Pract. Res. Clin. Rheumatol..

[5] Stolt P., Yahya A., Bengtsson C., Kallberg H., Ronnelid J., Lundberg I., Klareskog L., Alfredsson L., Group E.S. (2010). Silica exposure among male current smokers is associated with a high risk of developing ACPA-positive rheumatoid arthritis. Ann. Rheum. Dis..

[6] Semerano L., Julia C., Aitisha O., Boissier M.C. (2017). Nutrition and chronic inflammatory rheumatic disease. Joint Bone Spine.

[7] Daien C., Czernichow S., Letarouilly J.G., Nguyen Y., Sanchez P., Sigaux J., Beauvais C., Desouches S., Le Puillandre R., Rigalleau V. (2022). Dietary recommendations of the French Society for Rheumatology for patients with chronic inflammatory rheumatic diseases. Joint Bone Spine.

[8] Burdge G.C., Calder P.C. (2005). Conversion of alpha-linolenic acid to longer-chain polyunsaturated fatty acids in human adults. Reprod. Nutr. Dev..

[9] Harnack K., Andersen G., Somoza V. (2009). Quantitation of alpha-linolenic acid elongation to eicosapentaenoic and docosahexaenoic acid as affected by the ratio of n6/n3 fatty acids. Nutr. Metab..

[10] Kapoor R., Huang Y.S. (2006). Gamma linolenic acid: An antiinflammatory omega-6 fatty acid. Curr. Pharm. Biotechnol..

[11] de Pablo P., Romaguera D., Fisk H.L., Calder P.C., Quirke A.M., Cartwright A.J., Panico S., Mattiello A., Gavrila D., Navarro C. (2018). High erythrocyte levels of the n-6 polyunsaturated fatty acid linoleic acid are associated with lower risk of subsequent rheumatoid arthritis in a southern European nested case-control study. Ann. Rheum. Dis..

[12] Serhan C.N., Yang R., Martinod K., Kasuga K., Pillai P.S., Porter T.F., Oh S.F., Spite M. (2009). Maresins: Novel macrophage mediators with potent antiinflammatory and proresolving actions. J. Exp. Med..

[13] Saini R.K., Keum Y.S. (2018). Omega-3 and omega-6 polyunsaturated fatty acids: Dietary sources, metabolism, and significance—A review. Life Sci..

[14] Grimble R.F., Howell W.M., O’Reilly G., Turner S.J., Markovic O., Hirrell S., East J.M., Calder P.C. (2002). The ability of fish oil to suppress tumor necrosis factor alpha production by peripheral blood mononuclear cells in healthy men is associated with polymorphisms in genes that influence tumor necrosis factor alpha production. Am. J. Clin. Nutr..

[15] Moghaddami N., Irvine J., Gao X., Grover P.K., Costabile M., Hii C.S., Ferrante A. (2007). Novel action of n-3 polyunsaturated fatty acids: Inhibition of arachidonic acid-induced increase in tumor necrosis factor receptor expression on neutrophils and a role for proteases. Arthritis Rheum..

[16] Gutierrez S., Svahn S.L., Johansson M.E. (2019). Effects of Omega-3 Fatty Acids on Immune Cells. Int. J. Mol. Sci..

[17] Sigaux J., Mathieu S., Nguyen Y., Sanchez P., Letarouilly J.G., Soubrier M., Czernichow S., Flipo R.M., Sellam J., Daien C. (2022). Impact of type and dose of oral polyunsaturated fatty acid supplementation on disease activity in inflammatory rheumatic diseases: A systematic literature review and meta-analysis. Arthritis Res. Ther..

[18] Tanski W., Swiatoniowska-Lonc N., Tabin M., Jankowska-Polanska B. (2022). The Relationship between Fatty Acids and the Development, Course and Treatment of Rheumatoid Arthritis. Nutrients.

[19] Shapiro J.A., Koepsell T.D., Voigt L.F., Dugowson C.E., Kestin M., Nelson J.L. (1996). Diet and rheumatoid arthritis in women: A possible protective effect of fish consumption. Epidemiology.

[20] Rosell M., Wesley A.M., Rydin K., Klareskog L., Alfredsson L., EIRA Study Group (2009). Dietary fish and fish oil and the risk of rheumatoid arthritis. Epidemiology.

[21] Di Giuseppe D., Wallin A., Bottai M., Askling J., Wolk A. (2014). Long-term intake of dietary long-chain n-3 polyunsaturated fatty acids and risk of rheumatoid arthritis: A prospective cohort study of women. Ann. Rheum. Dis..

[22] Benito-Garcia E., Feskanich D., Hu F.B., Mandl L.A., Karlson E.W. (2007). Protein, iron, and meat consumption and risk for rheumatoid arthritis: A prospective cohort study. Arthritis Res. Ther..

[23] Pedersen M., Stripp C., Klarlund M., Olsen S.F., Tjonneland A.M., Frisch M. (2005). Diet and risk of rheumatoid arthritis in a prospective cohort. J. Rheumatol..

[24] Dourado E., Ferro M., Sousa Guerreiro C., Fonseca J.E. (2020). Diet as a Modulator of Intestinal Microbiota in Rheumatoid Arthritis. Nutrients.

[25] Hahn J., Cook N.R., Alexander E.K., Friedman S., Walter J., Bubes V., Kotler G., Lee I.M., Manson J.E., Costenbader K.H. (2022). Vitamin D and marine omega 3 fatty acid supplementation and incident autoimmune disease: VITAL randomized controlled trial. BMJ.

[26] Gan R.W., Young K.A., Zerbe G.O., Demoruelle M.K., Weisman M.H., Buckner J.H., Gregersen P.K., Mikuls T.R., O’Dell J.R., Keating R.M. (2016). Lower omega-3 fatty acids are associated with the presence of anti-cyclic citrullinated peptide autoantibodies in a population at risk for future rheumatoid arthritis: A nested case-control study. Rheumatology.

[27] Gan R.W., Demoruelle M.K., Deane K.D., Weisman M.H., Buckner J.H., Gregersen P.K., Mikuls T.R., O’Dell J.R., Keating R.M., Fingerlin T.E. (2017). Omega-3 fatty acids are associated with a lower prevalence of autoantibodies in shared epitope-positive subjects at risk for rheumatoid arthritis. Ann. Rheum. Dis..

[28] Gan R.W., Bemis E.A., Demoruelle M.K., Striebich C.C., Brake S., Feser M.L., Moss L., Clare-Salzler M., Holers V.M., Deane K.D. (2017). The association between omega-3 fatty acid biomarkers and inflammatory arthritis in an anti-citrullinated protein antibody positive population. Rheumatology.

[29] Lee A.L., Park Y. (2013). The association between n-3 polyunsaturated fatty acid levels in erythrocytes and the risk of rheumatoid arthritis in Korean women. Ann. Nutr. Metab..

[30] Combe B., Benessiano J., Berenbaum F., Cantagrel A., Daures J.P., Dougados M., Fardellone P., Fautrel B., Flipo R.M., Goupille P. (2007). The ESPOIR cohort: A ten-year follow-up of early arthritis in France: Methodology and baseline characteristics of the 813 included patients. Joint Bone Spine.

[31] Fezeu L.K., Laporte F., Kesse-Guyot E., Andreeva V.A., Blacher J., Hercberg S., Galan P. (2014). Baseline plasma fatty acids profile and incident cardiovascular events in the SU.FOL.OM3 trial: The evidence revisited. PLoS ONE.

[32] Assmann K.E., Lassale C., Andreeva V.A., Jeandel C., Hercberg S., Galan P., Kesse-Guyot E. (2015). A Healthy Dietary Pattern at Midlife, Combined with a Regulated Energy Intake, Is Related to Increased Odds for Healthy Aging. J. Nutr..

[33] Zhao J., Li Z., Gao Q., Zhao H., Chen S., Huang L., Wang W., Wang T. (2021). A review of statistical methods for dietary pattern analysis. Nutr. J..

[34] Rodriguez-Carrio J., Alperi-Lopez M., Lopez P., Ballina-Garcia F.J., Suarez A. (2016). Non-Esterified Fatty Acids Profiling in Rheumatoid Arthritis: Associations with Clinical Features and Th1 Response. PLoS ONE.

[35] Smits L.J., van Kuijk S.M., Leffers P., Peeters L.L., Prins M.H., Sep S.J. (2013). Index event bias-a numerical example. J. Clin. Epidemiol..

[36] Dahabreh I.J., Kent D.M. (2011). Index event bias as an explanation for the paradoxes of recurrence risk research. JAMA.

[37] Edefonti V., Parpinel M., Ferraroni M., Boracchi P., Schioppo T., Scotti I., Ubiali T., Currenti W., De Lucia O., Cutolo M. (2020). A Posteriori Dietary Patterns and Rheumatoid Arthritis Disease Activity: A Beneficial Role of Vegetable and Animal Unsaturated Fatty Acids. Nutrients.

[38] Baker D.G., Krakauer K.A., Tate G., Laposata M., Zurier R.B. (1989). Suppression of human synovial cell proliferation by dihomo-gamma-linolenic acid. Arthritis Rheum..

[39] Williams W.V., Rosenbaum H., Zurier R.B. (1996). Effects of unsaturated fatty acids on expression of early response genes in human T lymphocytes. Pathobiology.

[40] DeLuca P., Rossetti R.G., Alavian C., Karim P., Zurier R.B. (1999). Effects of gammalinolenic acid on interleukin-1 beta and tumor necrosis factor-alpha secretion by stimulated human peripheral blood monocytes: Studies in vitro and in vivo. J. Investig. Med..

[41] Dooper M.M., van Riel B., Graus Y.M., M’Rabet L. (2003). Dihomo-gamma-linolenic acid inhibits tumour necrosis factor-alpha production by human leucocytes independently of cyclooxygenase activity. Immunology.

[42] Veselinovic M., Vasiljevic D., Vucic V., Arsic A., Petrovic S., Tomic-Lucic A., Savic M., Zivanovic S., Stojic V., Jakovljevic V. (2017). Clinical Benefits of n-3 PUFA and -Linolenic Acid in Patients with Rheumatoid Arthritis. Nutrients.

[43] Reed G.W., Leung K., Rossetti R.G., Vanbuskirk S., Sharp J.T., Zurier R.B. (2014). Treatment of rheumatoid arthritis with marine and botanical oils: An 18-month, randomized, and double-blind trial. Evid. Based Complement. Alternat. Med..

[44] Gomez E.A., Colas R.A., Souza P.R., Hands R., Lewis M.J., Bessant C., Pitzalis C., Dalli J. (2020). Blood pro-resolving mediators are linked with synovial pathology and are predictive of DMARD responsiveness in rheumatoid arthritis. Nat. Commun..

[45] Sun Q., Ma J., Campos H., Hankinson S.E., Hu F.B. (2007). Comparison between plasma and erythrocyte fatty acid content as biomarkers of fatty acid intake in US women. Am. J. Clin. Nutr..

[46] Katan M.B., Deslypere J.P., van Birgelen A.P., Penders M., Zegwaard M. (1997). Kinetics of the incorporation of dietary fatty acids into serum cholesteryl esters, erythrocyte membranes, and adipose tissue: An 18-month controlled study. J. Lipid. Res..

[47] Salminen E., Heikkila S., Poussa T., Lagstrom H., Saario R., Salminen S. (2002). Female patients tend to alter their diet following the diagnosis of rheumatoid arthritis and breast cancer. Prev. Med..

[48] Hu X.F., Sandhu S.K., Harris W.S., Chan H.M. (2017). Conversion ratios of n-3 fatty acids between plasma and erythrocytes: A systematic review and meta-regression. Br. J. Nutr..

