# Supplementary material for: Serum Fatty Acid Profiles Are Associated with Disease Activity in Early Rheumatoid Arthritis: Results from the ESPOIR Cohort
Source: Nutrients. 2022 Jul 19;14(14):2947. doi: 10.3390/nu14142947 (PMC9322967; doi:10.3390/nu14142947)
Supplement: Supplementary file 1 [file nutrients-14-02947-s001.zip › nutrients-1792973-supplementary.pdf]

Supplementary material

Supplementary Table S1. Univariate analysis of the association between pattern ω6 and patient characteristics at inclusion in the cohort.

**Pattern ω6**		**T1**	**T2**	**T3**	**p**
Age, mean (SD)	--	47.0 (13.1)	47.4 (12.5)	51.3 (10.6)	**<0.001**
Smoking	Never smoker	132 (59.2)	123 (55.2)	100 (44.8)	**0.04**
	Former smoker	51 (22.9)	56 (25.1)	65 (29.2)	
	Current smoker	40 (17.9)	44 (19.7)	58 (26.0)	
BMI	Underweight or normal	149 (66.8)	128 (57.9)	114 (51.1)	**0.002**
	Overweight	57 (25.6)	59 (26.7)	64 (28.7)	
	Obesity	17 (7.6)	34 (15.4)	45 (20.2)	
Ethnicity	Other	21 (9.4)	16 (7.2)	17 (7.6)	0.66
	Caucasian	202 (90.6)	207 (92.8)	206 (92.4)	
Sex	Men	44 (19.7)	45 (20.2)	63 (28.3)	0.05
	Women	179 (80.3)	178 (79.8)	160 (71.8)	
Professional status	Working or student	140 (63.1)	140 (62.8)	127 (57.0)	0.68
	Unemployed (whatever the cause)	40 (18.0)	39 (17.5)	46 (20.6)	
	Retired	42 (18.9)	44 (19.7)	50 (22.4)	
Education level	Primary school	81 (36.3)	102 (45.7)	123 (55.2)	**<0.001**
	High school	51 (22.9)	48 (21.5)	53 (23.8)	
	Graduate education	91 (40.8)	73 (32.7)	47 (21.1)	
Marital status	Single	69 (31.1)	57 (25.6)	56 (25.1)	0.29
	Married	153 (68.9)	166 (74.4)	167 (74.9)	
ACPA	Positive	101 (45.7)	90 (40.7)	120 (54.8)	**0.01**
IgM-RF	Positive	132 (59.2)	115 (51.6)	138 (61.9)	0.07
Typical RA erosions	Yes	30 (13.5)	25 (11.2)	45 (20.2)	**0.02**
corticosteroids	Yes	44 (19.7)	41 (18.4)	36 (16.1)	0.61
NSAIDs	Yes	206 (92.4)	197 (88.3)	202 (90.6)	0.35
Beta-blockers	Yes	11 (4.9)	15 (6.7)	25 (11.3)	**0.03**
Statins	Yes	7 (3.1)	22 (9.9)	15 (3.8)	**0.02**
HRT or OC	Yes	55 (24.7)	29 (13.0)	28 (12.6)	**<0.001**
Baseline use of DMARDs	Yes	19 (8.5)	18 (9.1)	15 (6.7)	0.76
Baseline DAS28	> 5.1 (high)	114 (53.0)	94 (42.7)	132 (59.7)	**0.002**
CRP, mean (SD)	--	18.6 (28.1)	17.2 (27.5)	26.5 (43.0)	**0.007**

Data are n (%) unless indicated. Bold p values are significant (P<0.05). BMI: body mass index; CRP: C-reactive protein; DAS28: disease activity score in 28 joints based on ESR; HRT: hormonal replacement therapy; OC: oral contraception; RA: rheumatoid arthritis; ACPA: anti-citrullinated peptide antibodies; RF: rheumatoid factor (IgM); c/bDMARD: conventional/biological disease-modifying anti-rheumatic drugs; NSAIDs: non-steroidal anti-inflammatory drugs; T1, T2, T3: tertile 1, 2 and 3 of pattern score, respectively.

Supplementary Table S2. Univariate analysis of the association between pattern ω7-9 and patient characteristics at inclusion in the cohort

**Pattern ω7-9**		**T1**	**T2**	**T3**	**p**
Age, mean (SD)	--	46.4 (12.5)	48.7 (12.0)	50.6 (12.0)	**0.002**
Smoking	Never smoker	125 (56.3)	131 (58.5)	99 (44.4)	**0.03**
	Former smoker	55 (24.8)	51 (22.8)	66 (29.6)	
	Current smoker	42 (18.9)	42 (18.8)	58 (26.0)	
BMI	Underweight or normal	150 (67.9)	132 (58.9)	109 (49.1)	**<0.001**
	Overweight	56 (25.3)	50 (22.3)	74 (33.3)	
	Obesity	15 (6.8)	42 (18.8)	39 (17.6)	
Ethnicity	Other	28 (12.6)	20 (8.9)	6 (2.7)	**<0.001**
	Caucasian	194 (87.4)	204 (91.1)	217 (97.3)	
Sex	Men	41 (18.5)	50 (22.3)	61 (27.4)	0.08
	Women	181 (81.5)	174 (77.7)	162 (72.7)	
Professional status	Working or student	133 (59.9)	150 (67.0)	124 (55.9)	**0.046**
	Unemployed (whatever the cause)	50 (22.5)	32 (14.3)	43 (19.4)	
	Retired	39 (17.6)	42 (18.8)	55 (24.8)	
Education level	Primary school	101 (45.5)	100 (44.6)	105 (47.1)	0.83
	Highschool	53 (23.9)	47 (21.0)	52 (23.3)	
	Graduate education	68 (30.6)	77 (34.4)	66 (29.6)	
Marital status	Single	62 (27.9)	61 (27.2)	59 (26.6)	0.95
	Married	160 (72.1)	163 (72.8)	163 (73.4)	
ACPA	Positive	115 (52.3)	97 (43.9)	99 (45.0)	0.16
IgM-RF	Positive	133 (59.9)	125 (55.8)	127 (57.0)	0.66
Typical RA erosions	Yes	34 (15.3)	31 (13.8)	35 (15.8)	0.84
Corticosteroids	Yes	35 (15.8)	46 (20.5)	40 (17.9)	0.42
NSAIDs	Yes	194 (87.4)	203 (90.6)	208 (93.3)	0.11
Beta-blockers	Yes	9 (4.1)	20 (8.9)	22 (9.9)	**0.048**
Statins	Yes	8 (3.6)	14 (2.3)	22 (9.9)	**0.03**
HRT or OC	Yes	27 (12.2)	36 (16.1)	49 (22.0)	**0.02**
Baseline use of DMARDs	Yes	15 (6.8)	17 (7.6)	20 (9.0)	0.68
Baseline DAS28	> 5.1 (high)	102 (47.0)	114 (51.1)	124 (57.4)	0.09
CRP, mean (SD)	--	18.0 (29.9)	20.0 (36.8)	24.2 (34.3)	0.15

Data are n (%) unless otherwise indicated. Bold p values are significant (P<0.05).
BMI: body mass index; CRP: C-reactive protein; DAS28: disease activity score in 28 joints based on ESR; HRT: hormonal replacement therapy; OC: oral contraception; RA: rheumatoid arthritis; ACPA: anti-citrullinated peptide antibodies; RF: rheumatoid factor (IgM); c/bDMARD: conventional/biological disease-modifying anti-rheumatic drugs; NSAIDs: non-steroidal anti-inflammatory drugs; T1, T2, T3: tertile 1, 2 and 3 of pattern score, respectively.

Supplementary Table S3. Univariate analysis of the association between baseline FA patterns and 6-month high disease activity (DAS28 >5.1) (n=594)

	T1	T2	T3	p	p trend
**Pattern ω7-9**					
N (%) with DAS28 >5.1	31 (15.2)	28 (13.3)	25 (12.3)		
OR (95% CI)	Ref	0.81 (0.46-1.42)	0.61 (0.33-1.11)	0.69	0.4
**Pattern ω3**					
N (%) with DAS28 >5.1	38 (18.1)	26 (12.8)	20 (9.9)		
OR (95% CI)	Ref	0.66 (0.38-1.14)	0.49 (0.27-0.88)	0.05	0.01
**Pattern ω6**					
N (%) with DAS28 >5.1	39 (18.9)	19 (9.2)	26 (12.7)		
OR (95% CI)	Ref	0.43 (0.24-0.78)	0.62 (0.36-1.06)	0.01	0.06

DAS28: Disease Activity Score in 28 joints; OR: odds ratio; 95% CI: 95% confidence interval

Supplementary Table S4. Multivariate analysis of the association between FA patterns and 6-month moderate-to-high disease activity (DAS28 ≥ 3.2) (n= 594)

		T1	T2	T3	p trend
**Pattern ω7-9**					
OR (95% CI)	Model 1	Ref	0.78 (0.51-1.20)	1.03 (0.66-1.59)	0.90
	Model 2	Ref	0.78 (0.51-0.20)	1.01 (0.65-1.56)	0.97
**Pattern ω3**					
OR (95% CI)	Model 1	Ref	0.93 (0.60-1.44)	0.72 (0.45-1.14)	0.15
	Model 2	Ref	0.95 (0.61-1.48)	0.74 (0.47-0.17)	0.19
**Pattern ω6**					
OR (95% CI)	Model 1	Ref	0.98 (0.64-1.50)	0.95 (0.61-1.47)	0.80
	Model 2	Ref	0.97 (0.64-1.49)	0.92 (0.59-1.42)	0.71

Model 1: adjusted for age, sex, BMI, smoking status, education level, work, baseline ACPA status, baseline RF status, baseline DAS28, baseline CRP level, baseline treatments (NSAIDs, corticosteroids, statins, HRT or OC, beta blockers, DMARDs treatment between 0 and 6 months. Model 2: model 1 not adjusted for baseline CRP level

BMI: body mass index; CRP: C-reactive protein; HRT: hormone replacement therapy; OC: oral contraception; ACPA: anti-citrullinated peptide antibodies; RF: rheumatoid factor (IgM); NSAIDs: nonsteroidal anti-inflammatory drugs; OR: odds ratio; 95% CI: 95% confidence interval

Supplementary Table S5. Multivariate analysis of the association between FA patterns and 6-month non-remission (DAS28 ≥ 2.6) (n=594)

		T1	T2	T3	p trend
**Pattern ω7-9**					
OR (95% CI)	Model 1	Ref	0.74 (0.47-1.17)	1.02 (0.63-1.64)	0.98
	Model 2	Ref	0.74 (0.47-1.17)	1.00 (0.62-1.62)	0.98
**Pattern ω3**					
OR (95% CI)	Model 1	Ref	0.99 (0.61-1.60)	0.63 (0.38-1.04)	0.07
	Model 2	Ref	1.00 (0.62-1.63)	0.65 (0.39-1.06)	0.08
**Pattern ω6**					
OR (95% CI)	Model 1	Ref	1.06 (1.68-1.67)	0.95 (0.59-1.53)	0.85
	Model 2	Ref	1.06 (0.67-1.66)	0.92 (0.58-1.48)	0.76

Model 1: adjusted for age, sex, BMI, smoking status, education level, work, baseline ACPA status, baseline RF, baseline DAS28, baseline CRP level, baseline treatments (NSAIDs, corticosteroids, statins, HRT or OC, beta blockers, DMARDs treatment between 0 and 6 months. Model 2: model 1 not adjusted for baseline CRP level

BMI: body mass index; CRP: C-reactive protein; HRT: hormone replacement therapy; OC: oral contraception; ACPA: anti-citrullinated peptide antibodies; RF: rheumatoid factor (IgM); NSAIDs: nonsteroidal anti-inflammatory drugs; OR: odds ratio; 95% CI: 95% confidence interval
